# Skin and fat necrosis of the breast following methylene blue dye injection for sentinel node biopsy in a patient with breast cancer

**DOI:** 10.1186/1477-7800-2-26

**Published:** 2005-11-28

**Authors:** M Salhab, W Al sarakbi, K Mokbel

**Affiliations:** 1 St Georges and The Princess Grace Hospitals, London, UK

**Keywords:** Sentinel lymph node, methylene blue dye, breast cancer, skin necrosis

## Abstract

Sentinel lymph node biopsy (SLNB) is a simple technique that uses subdermal or peri-tumoral injection of vital blue dye and/or radioactive isotope to identify the first lymph node(s) draining the primary tumor. It has been shown to accurately predict axillary node status in patients with clinically node negative breast cancer. The SLNB is emerging as a new standard of care in patients with early breast cancer. However, the use of methylene blue (MB) dye can be associated with a number of local complications due to its tissue reactive properties. We report a rare case of skin and fat necrosis followed by a dry gangrene of the skin in a female patient with breast cancer who underwent SLNB localization using peri-tumoral injection of MB dye in another institution. This case and literature review suggest that the use of MB dye for SLNB identification should be avoided and replaced with alternative types of blue dye such as Patent Blue V preferably in conjunction with a radioactive isotope tracer.

## Introduction

Sentinel lymph node biopsy (SLNB) is emerging as a new standard in the treatment of patients with operable breast cancer. It is a simple technique that uses subdermal or peri-tumoral injection of vital blue dye and/or radioactive isotope to identify the first lymph node(s) draining the primary tumor. SLNB is a reliable and minimally invasive procedure, which accurately predicts axillary node status in patients with clinically node negative breast cancer [1]. Localization of the sentinel lymph node using the intradermal, subareolar or peritumoural injection of a vital blue dye is widely practiced [2]. However, local and systemic complications secondary to the use of the dye have been reported.

We report a rare case of severe skin and fat necrosis secondary to the injection of methylene blue dye in a patient with T1 breast cancer. This complication occurred in a different institution.

## Case report

A postmenopausal woman was referred to the senior author (KM) following breast conserving surgery for 17 mm invasive ductal carcinoma of the right breast. Localization of the sentinel lymph node was performed using the dual localization technique. Intradermal injection of technetium-labled sulphur colloid was performed over the tumour site. The following day, the patient had the MB dye injected in the peritumoral area. The operation was uneventful.

**Figure 1:**
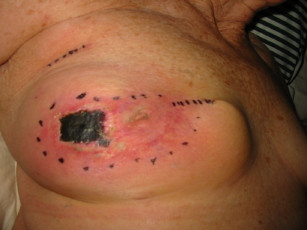
Skin and fat necrosis of the right breast secondary to injection of methylene blue dye for SLNB.

Three days post operatively and upon removal of the wound dressing, the lateral aspect of the breast skin exhibited a rectangular erythematous violaceous surface which developed into a dry gangrenous area a few days later (Figure 1). We believe that the patient developed skin and fat necrosis secondary to the MB dye injection. This complication may have been caused by a localised tissue reaction initiated by, or involving the dye.

## Discussion

The technique of blue dye mapping was first described for breast cancer by Giuliano et al [3]. Isosulfan blue dye has been traditionally used the dye used for SLNB for breast cancer. However, its use was associated with a significant number of allergic reactions [4], some of which are life threatening. Because methylene blue dye has been shown to be equally effective and does not pose a serious risk of severe allergic and hypersensitivity reactions, it was regarded as an acceptable substitute for isosulfan blue dye for SLNB [5-8]. Although, the use of the MB dye for SLNB in breast cancer has fewer allergic reactions, its use has been associated with a number of local and systematic complications. Stradling et al, was the first to report adverse skin reactions to methylene blue dye in patients with breast cancer [9]. In addition, skin eruptions and rashes [10], subcutaneous tissue necrosis and abscess formation [11] have been reported in association with the injection of this dye. Furthermore, capsular contraction following breast reconstruction using an implant with intense blue discoloration of the prosthesis was reported in a patient in whom methylene blue dye was used to identify the sentinel lymph node [12].

In our reported case, severe skin and fat necrosis complicated the peri-tumoral injection of methylene blue dye; This might be due to that methylene blue dye may induce an early foreign body-type reaction characterized by ischemic ulceration, fibrinoid necrosis with eosinophilic infiltration [13].

Therefore, we recommend the use of Patent Blue V dye instead of MB for SLNB localization in patients with breast cancer in order to avoid such significant complications which may delay subsequent treatment. Patent Blue dye has been reported to cause minor local complications in form of long-term discoloring of the skin at the site of injection [14]. Although no cases of severe local tissue necrosis has been reported in association with Patent Blue V dye, however, anaphylactic shock has been observed following its injection for SLNB localization [15,16]. The risk of allergic reactions can be reduced by using corticosteroids and antihistamines [4,17,18]

In conclusion, the use of MB dye for SLNB identification should be avoided and replace with alternative types of blue dye such as Patent Blue V preferably in conjunction with a radioactive isotope tracer.
